# Targeting the IL-17A pathway for therapy in early-stage tendinopathy

**DOI:** 10.1136/rmdopen-2024-004729

**Published:** 2025-02-23

**Authors:** Neal L Millar, Iain B McInnes, Frank Kolbinger, Friedrich Raulf, Moeed Akbar, Yufei Li, Nicolau Beckmann, Nathalie Accart, Olivier Leupin, Claudio Calonder, Matthias Schieker, Michaela Kneissel, Christian Bruns, Richard M Siegel, Eckhard Weber

**Affiliations:** 1 School of Infection and Immunity, University of Glasgow, Glasgow, UK; 2 Novartis Pharma AG, Biomedical Research, Basel, Switzerland

**Keywords:** Interleukin-17, Tendinopathy, Inflammation

## Abstract

**Objectives:**

Tendinopathy is a frequent clinical problem and represents an extraordinary health economic and socioeconomic burden with high unmet medical needs. Recent clinical evidence suggests blockade of interleukin 17A (IL-17A) for tendinopathy therapy. The present preclinical study elucidates the biological mechanisms of IL-17A pathway stimulation and blockade in tendinopathy.

**Methods:**

We explored whether IL-17A and other IL-17 family members are differentially expressed in biopsies of healthy, early-stage and late-stage tendinopathic human rotator cuff tendons using RT-qPCR. IL-17 pathway signature genes in healthy human tendon-derived cells were identified following IL-17A stimulation using AmpliSeq RNA. The molecular, structural and functional consequences of IL-17A pathway stimulation were explored in healthy human tendon-derived cells and in a rat tendon fascicle model ex vivo. The effects of IL-17A pathway blockade were investigated in a rat model of rotator cuff tendinopathy in vivo.

**Results:**

We provide evidence of differential expression of IL-17A mRNA (*IL17A*) versus other IL-17 family members in human rotator cuff early-stage tendinopathy. In human tendon-derived cells, stimulation with IL-17A induced the expression of the selected IL-17A pathway signature genes *NFKBIZ, ZC3H12A, CXCL1, IL6, MMP3*. Expression was inhibited by IL-17A blockade. In the rat ex vivo and in vivo models, IL-17A blockade alleviated inflammatory immune effector release, tendon structural degeneration, tendon inflammation and impaired tendon function.

**Conclusion:**

Our data provide evidence that IL-17A is a key contributor to the pathogenesis of tendinopathy by promoting tendon inflammation and degeneration and that IL-17A blockade may represent a potential therapy in early-stage tendinopathy.

**WHAT IS ALREADY KNOWN ON THIS TOPIC**

- Recent findings in cellular systems suggest that interleukin (IL)-17A contributes to the pathogenesis of human tendinopathy by promoting tendon inflammation and tendon matrix degeneration. A first clinical Ph2 trial with the anti-IL17A mAb secukinumab in patients with rotator cuff tendinopathy (NCT03344640) revealed clinically relevant and significant improvement of tendinopathic symptoms in a patient population with early-stage moderate-to-severe tendinopathy identified by prespecified post hoc analysis.

**WHAT THIS STUDY ADDS**

- Here, we provide mechanistic evidence of the molecular, structural and functional consequences of IL-17A pathway modulation in preclinical tendon pathobiology models. We confirm the differential expression of *IL17A* in rotator cuff tendon biopsies obtained from patients with early-stage tendinopathy. We demonstrate that blockade of IL-17A prevents degenerative disorganisation of tendon extracellular matrix, improves mechanical function of isolated rat tendon fascicles, resolves tendon inflammation, reverses tendon swelling and attenuates impaired tendon mechanics and deficits in shoulder function in a rat rotator cuff tendon injury model.

**HOW THIS STUDY MIGHT AFFECT RESEARCH, PRACTICE OR POLICY**

- The present study provides evidence that IL-17A blockade interferes with the pathobiology of tendinopathy and may be a potential therapy in early-stage tendinopathy.

## Introduction

 Tendinopathy represents a largely underestimated group of musculoskeletal conditions associated with chronic inflammation of the tendon and dysregulated tendon tissue regeneration.^1^ It accounts for 30%–50% of all sporting injuries and a high proportion of rheumatological and orthopaedic referrals from primary care physicians.^2^ The health economic and socioeconomic impact of tendinopathy is high and similar to other chronic musculoskeletal conditions, such as knee osteoarthritis and rheumatoid arthritis.^3^

The pathogenesis of tendinopathy is thought to be elicited by persistent mechanical microinjury and inappropriate resolution of injury-induced inflammation due to dysregulated cross-talk of immune cells and tendon stromal cells.^4 5^ Recent advances in single-cell and spatial transcriptomics of human tendon have revealed the dysregulated immune homeostasis in tendinopathy^6^ and identified clusters of various human tendon cell types in health and disease, embracing immune cells and stromal cells including tenocytes, tendon resident stem/progenitor cells and endothelial cells.^6^,^8^ Cytokines have been implicated as early immune effectors in tendinopathy, and expression of inflammatory cytokines and associated immune cell infiltration is most pronounced in the early stages of tendinopathy.^9^ In rotator cuff tendon biopsies of early-stage human tendinopathy, we have previously shown an increased expression of IL-17A mRNA (*IL17A*) and the presence of IL-17A protein in mast cells, macrophages and T cells, pointing towards a pathogenetic role of IL-17A in tendinopathy.^10^

It has been proposed that tendinopathy shares pathogenetic commonalities with enthesitis in response to microinjury,^11 12^ and the presence of immune cells including neutrophils, T cells, and myeloid cells is common in both disease entities.^13^ In enthesitis, IL-17A is expressed by immune cells and is thought to augment the injury-induced inflammation through the release of cytokines and other mediators by stromal cells, which in turn trigger neutrophil migration and activation.^13^ Indeed, IL-17A blockade has been shown to be therapeutically effective in enthesitis, a characteristic clinical manifestation in spondyloarthritis and psoriatic arthritis.^14 15^ The overlap in the pathogenesis of tendinopathy and enthesitis, along with the previously identified potential role for IL-17A in human tendinopathy, inspired the conduct of a clinical Ph2 study to investigate the anti-IL-17A mAb secukinumab as a novel therapeutic approach for rotator cuff tendinopathy (NCT03344640). This study—while it failed its primary endpoint in the broad study population encompassing patients with early-stage and late-stage tendinopathy—revealed significant and clinically relevant improvement of tendinopathic symptoms by IL-17A blockade in the patient population with early-stage moderate-to-severe rotator cuff tendinopathy as identified by prespecified post hoc analysis.^16^

The present preclinical study aimed first to elucidate whether in addition to IL-17A, there is differential expression of its receptor subunits, IL-17RA and IL-17RC, and the other five IL-17 cytokine family members in human tendinopathy, second to provide evidence for the molecular, structural and functional consequences of IL-17A pathway activation in human tendon-derived cells and in rat tendon fascicles ex vivo, and finally to investigate the effects of IL-17A blockade in a rat model of rotator cuff tendinopathy in vivo, for further elucidation of the biological mechanisms underlying the reported clinical symptom relief by secukinumab in human early-stage tendinopathy.

## Methods

### Human tendon tissue collection and cell preparation

Supraspinatus tendon samples were collected from 10 patients with rotator cuff tears undergoing shoulder surgery. The mean tear size was 2.5 cm^2^. The supraspinatus samples showed a Bonar grade 4, consistent with marked degeneration, mucoid change and frank chondroid metaplasia, representing late-stage tendinopathy.^10^ Samples of the adjacent subscapularis tendon were collected from the same patients. The subscapularis tendon showed Bonar grades 2–3, representing early-stage tendinopathy.^10^ The tendinopathy patients were included according to the following criteria: a history of shoulder pain and dysfunction, no previous surgery on the affected shoulder, no radiographic sign of fracture of the shoulder and no history of rheumatoid arthritis or osteoarthritis. The age of the tendinopathy patients ranged from 35 to 70 years. Dedicated control samples of the subscapularis tendon were collected from six subjects undergoing arthroscopic surgery for shoulder stabilisation without rotator cuff tears. Subjects were only included if there was no clinically detectable evidence of subscapularis tendinopathy on a preoperative MRI scan, no macroscopic damage to the subscapularis tendon at arthroscopic examination, no previous shoulder surgery, no radiographic signs of shoulder fracture and no history of rheumatoid arthritis or osteoarthritis. All subscapularis tendon-derived control samples were classified as Bonar grade 1 consistent with normal fibrotendinous tissue with large distinct collagen fibrils.^10^ The age of the control group subjects ranged from 20 to 41 years.

Arthroscopic repair of the rotator cuff supraspinatus tendon tear was carried out using the standard three-portal technique while the cross-sectional size of the tear was estimated and recorded as previously described.^17^ The supraspinatus tendon was harvested from within 1.5 cm of the tear’s edge before surgical repair. The adjacent subscapularis tendon was biopsied arthroscopically from the superior border of the tendon 1 cm lateral to the glenoid labrum representing mid-body tendon structure.

Human tendon-derived cells were obtained from surgical surplus healthy hamstring tendons (n=8) and gracilis tendons (n=3) of 11 subjects undergoing tendon anterior cruciate ligament reconstruction. Healthy control tendons of different anatomical origin are commonly used for mechanistic cell experiments,^6 7^ assuming that the cell population in the midsubstance of healthy tendons is comparable across anatomical regions. The age of the subjects ranged from 18 to 30 years. Tendon midsubstance tissue was cut into small pieces with a scalpel and transferred into flasks with complete RPMI (RPMI media supplemented with 10% FBS, penicillin 100 I.U./mL, streptomycin 100 µg/mL), which were placed in an incubator at 37°C, 5% CO_2_. Cultures were maintained in RPMI medium at 37 °C in a humidified atmosphere of 5% CO_2_ for 28 days. The tendon-derived cells were subcultured and trypsinised at subconfluency, with cells from the third and fourth passage used.

### Stimulation of human tendon-derived cells

Tendon-derived cells were seeded into 24 well tissue culture plates (25 000 cells/well) and left to rest for 3 days at 37°C, 5% CO_2_. On day 3, media was replaced with fresh complete RPMI, and cells were stimulated with 10 or 100 ng/mL human IL-17A (Biolegend, UK), 0.15 ng/mL human TNF (Biolegend, UK), or control media (N2) in presence or absence of 100 µg/mL human anti-IL-17A IgG1/κ monoclonal antibody (mAb) secukinumab (Novartis, Switzerland) or inactive control IgG mAb (Biolegend, UK). After 24 hours, media was removed and 300 µL of RLT cell lysis buffer (Life Technologies, UK) was added to each well. Cell lysates were stored at −20°C for subsequent assessment of mRNA expression. Protein concentration of IL-6 was determined using an ELISA kit as per the manufacturer’s instruction (Invitrogen, UK).

### Human gene expression profiling by real-time qPCR

Tendon tissues for RNA analysis were placed in RNAlater (Ambion, UK) at the time of surgery. Prior to mRNA extraction, tendon tissues and tendon-derived cells (harvested separately) that were used for subsequent experiments were placed in Trizol. QIAgen mini columns which included an on-column DNase step (Qiagen, UK) were used for the RNA purification as per manufacturer’s instruction. cDNA was prepared from RNA samples using the AffinityScript multiple temperature cDNA synthesis kit (Agilent Technologies, USA) as per manufacturer’s instruction. Quantification of RT qPCR was performed using SYBR green mastermix (Applied Biosystems, USA). Before setting up the SYBR green assay, the cDNA was diluted one in five using RNase-free water. Each sample was analysed in triplicate. The relative expression of the mRNA of the gene of interest was calculated by the ΔCT method relative to the GAPDH housekeeping gene.

### Human gene expression profiling by AmpliSeq RNA

Total RNA was isolated by RNeasy kit including DNase digestion (Qiagen, UK). RNA was quantified by NanoDrop 1000 spectrophotometry (Thermo Fisher Scientific, UK), and RNA quality was assessed using a Bioanalyzer 2100 (Agilent, USA). Reverse transcription of 10 ng total RNA, amplification of 20 802 targets by ultra-high multiplex PCR and sequencing library construction were performed using the Ion AmpliSeq Transcriptome Human Gene Expression Kit (#A26325, Thermo Fisher Scientific, UK) and Ion Xpress barcodes. Unamplified libraries were quantified by qPCR and equal amounts were combined for sequencing. Templating of Ion Sphere Particles by emulsion PCR was conducted using the Ion 540 Kit (#A27753, Thermo Fisher Scientific, UK) and an OT2 instrument. Sequencing using an Ion 540 semiconductor chip was carried out on the Ion GeneStudio S5 System (Thermo Fisher Scientific, UK). Initial analysis comprising quality control and normalisation of mapped reads as RPM (reads per million mapped reads) was performed by the AmpliSeqRNA plug-in on the Ion Torrent server.

### Rat tail tendon fascicle model ex vivo

#### Tendon fascicle assay

Rats were sacrificed with CO_2_ and entire tails were harvested and kept on ice. Two 4 cm long segments were dissected from the proximal part of the tail with the skin remaining intact. Tendon fascicles were gently drawn from the tail segments using forceps. The fascicles were washed with sterile Dulbecco’s modified Eagle medium with nutrient mixture F12 (DMEM/F12, Gibco #31331093, Thermo Fisher Scientific, Switzerland) and were then tested immediately or were submitted to a tendon fascicle degeneration assay.

Progressive structural degeneration of the isolated tendon fascicles was induced by culturing them in the absence of mechanical loading. Pairs of fascicles were cultured free-floating in wells containing 2 mL serum-free standard tissue culture medium composed of DMEM/F12, N2 supplement (1% v/v, Gibco #17502048, Thermo Fisher Scientific, Switzerland), L(+)-ascorbic acid (300 µg/mL, Wako Chemicals, USA) and Pen-strep (1% v/v, Gibco #15140122, Thermo Fisher Scientific, Switzerland) for up to 10 days at 37°C. The culture medium was replaced at day 7.

The time-dependent effects of recombinant rat IL-17A (30 ng/mL, #8410-IL-025/CF, R&D systems, USA) and vehicle (phosphate buffered saline, PBS) on tendon fascicle degeneration were assessed at days 3, 7, and 10 of culture and compared with non-degenerated freshly isolated tendon fascicles. Recombinant rat IL-17A and vehicle were added to the culture medium at the beginning of culture and on day 7 to the replaced culture medium. The concentration-dependent effects of IL-17A (0, 0.3, 3, 30 ng/mL) on tendon fascicle degeneration were assessed at day 10 of culture and compared with non-degenerated freshly isolated tendon fascicles. The effects of blockade of IL-17A were assessed using a Novartis proprietary rodent-cross-reactive potent and selective low-molecular-weight IL-17A antagonist (IC_50_=10 ng/mL or 16 nM), premixed in culture medium at a concentration of 1 µM with the highest concentration of IL-17A (30 ng/mL). The culture medium was replaced at day 7.

#### Tendon fascicle read-outs

##### Tendon fascicle cytokine and chemokine release

Supernatants were collected from the tendon fascicle culture at day 10, and the concentration of the cytokine IL-6 and the chemokine CXCL1 (KC/GRO) in the supernatant was assessed with multiplex ELISA (V-PLEX Proinflammatory Panel 2 Rat Kit, K15059D, Meso Scale Discovery, USA) as per manufacturer’s instruction.

##### Tendon fascicle histopathology

Tendon fascicles were fixed with 10% (v/v) neutral buffered formalin for 4 hours and then embedded in paraffin. The tendon fascicles were cut along the longitudinal axis into slices of 5 µm thickness. Slices obtained from the core of the tendon fascicles were mounted and stained with Alcian blue (A-3157, Sigma, Switzerland) and Nuclear Fast Red (N-8002, Sigma, Switzerland). Stained slides were scanned with an Aperio slide scanner (Leica Biosystems, Germany) for qualitative histopathology assessment.

##### Tendon fascicle mechanics

Tail tendon fascicle structural degeneration was also assessed as fascicle mechanical function at day 10 of free-floating culture in comparison with the mechanical function of non-degenerated freshly isolated tendon fascicles using a material load-displacement test device (ElectroPuls E3000, 50 N load-cell, Instron, France) equipped with a customised organ bath chamber to immerse clamped fascicles in PBS during mechanical testing. Fascicles were preloaded to a position where crimp disappeared, and initial length (L0) based on grip-to-grip distance was recorded. Images of the fascicle were taken from orthogonal perspectives using two telecentric lenses (FABRIMEX T80 1.0L, Fabrimex AG, Switzerland) to characterise the ellipsoidal cross-sectional area of each fascicle specimen. Applying a standardised force-displacement (stress-strain) protocol, tendon fascicles were ramped to failure stress at a constant strain rate of 0.025% L0/s. Sample stress (load in N) and the corresponding strain (displacement in mm) were recorded to calculate Young’s modulus from the linear region of a stress-strain curve. Failure stress was determined as the stress threshold causing tendon fascicle rupture.

### Rat rotator cuff tendinopathy model in vivo

#### Rotator cuff supraspinatus tendon injury

Adult female Sprague Dawley rats were used (age: >6 months; body weight: 300–400 g, Janvier, France). Unilateral rotator cuff supraspinatus tendon injury was performed aseptically under general anaesthesia with isoflurane. A skin incision was made from the acromion towards the humerus bone and the deltoid muscle was split to expose the rotator cuff. The supraspinatus tendon was then pulled from the subacromial space with spinal cord hooks and kept under moderate tension. To create the full thickness, full length supraspinatus split tenotomy, a 23G cannula was positioned along the long axis of the supraspinatus tendon to guide cutting using a scalpel. The deltoid muscle and the skin were subsequently closed layer-by-layer. After recovery from anaesthesia, rats returned to cage activity and postoperative pain management was maintained with buprenorphine (0.05 mg/kg, sc, Temgesic, Eumedica, Switzerland) twice daily for 3 days.

#### Rotator cuff tendinopathy study design

The effects of pharmacological IL-17A blockade on supraspinatus tendon injury-induced rotator cuff inflammation and impaired gait were assessed serially in a randomised vehicle-controlled 4-week study. For blockade of IL-17A, a Novartis proprietary rodent-cross-reactive anti-IL-17A IgG1/κ mAb was used. Rats were dosed either with the anti-IL-17A mAb (15 mg/kg, sc) or with vehicle (PBS 0.25 mL/kg, sc) once a week for 4 weeks with the first dose administered 5 days prior to tendon injury. The anti-IL-17A mAb exhibited terminal half-life of more than 2 weeks resulting in pharmacologically relevant serum levels of >60 µg/mL throughout the entire experiment. The body weight of anti-IL-17A mAb and vehicle-treated rats was similar and did not substantially change in the course of the study.

#### Rotator cuff tendinopathy read-outs

##### Inflammation assessment

Anti-inflammatory effects of IL-17A blockade following rotator cuff supraspinatus tendon injury were assessed by performing serial MRI examinations with a Pharmascan 7 Tesla scanner (Bruker Medical Systems, Germany) under general anaesthesia with isoflurane at baseline prior to injury and at day 7 and day 21 postinjury. Sagittal and axial images were acquired using a Turbo-RARE sequence (effective echo time 21 ms, repetition time 5600 ms, RARE factor 4, field-of-view 4×3 cm (sagittal orientation) or 3×4 cm (axial orientation), matrix 256×192, slice thickness 0.4 mm, 30 slices). Changes in the normalised T_2_-weighted signal around the injured supraspinatus tendon over time were measured to assess anti-inflammatory effects of IL-17A blockade compared with vehicle treatment.

##### Gait analysis

Effects on mobility and shoulder function of IL-17A blockade following rotator cuff supraspinatus tendon injury were assessed by gait analysis (CatWalk XT 10, Noldus, The Netherlands). Gait was recorded and analysed at baseline prior to injury and in weeks 1, 2 and 4 postinjury. Following habituation for three subsequent days, rats were trained to traverse a walkway on a glass plate with a speed between 50 and 100 cm/s and the footprints of their paws were captured by a high-speed video camera positioned underneath the walkway. Three runs per trial were collected. The paw maximum contact area (cm^2^) and maximum intensity at maximum contact (arbitrary unit) were assessed for each individual paw. For gait analysis, the relative contribution to contact area and intensity of the injured left front paw and the right hind paw were calculated in accordance with the diagonal weight-bearing locomotion trot of rats.

##### Supraspinatus tendon mechanics

Effects on tendon function of IL-17A blockade following rotator cuff supraspinatus tendon injury were terminally assessed by mechanics of the excised supraspinatus tendon. Rats were sacrificed with CO_2_ at day 28 postinjury and the injured and healthy contralateral shoulders were collected, wrapped in PBS-soaked gauze, and stored at −20°C. For mechanical testing, frozen specimens were thawed to room temperature, rehydrated in PBS and a precise anatomical preparation of the supraspinatus tendon attached to the humerus bone was generated. The geometric dimensions of the supraspinatus tendon were determined in situ with a digital slide calliper to calculate the cross-sectional area. The mechanical tendon function was assessed using a standard uniaxial material load-displacement test device (ElectroPuls E3000, 250 N load-cell, Instron, France). The supraspinatus tendon preparation was mounted into the test device using a customer-tailored stainless steel clamping system positioned in a PBS-filled chamber at room temperature. Tensile force was applied along the longitudinal axis of the supraspinatus tendon, positioned in 45° of abduction relative to the humerus.

The mechanical test protocol contained three steps. First, the supraspinatus tendon preparation was preconditioned to minimise mechanical hysteresis and viscoelastic creep by applying five load-unload cycles at 0.5 Hz and at 2.5% strain of the tendon’s original length (L0). Second, a physiological low-load dynamic frequency test at 0.1, 1, 5 and 10 Hz, for 10 cycles each was performed with a maximum strain of 0.25% of L0 using a sinusoidal waveform. Finally, the supraspinatus tendon was unloaded and then subjected to ultimate failure testing. The transmitted stress (load in N) and strain (displacement in mm) of the supraspinatus tendon were measured simultaneously, and the dynamic modulus (MPa) and tensile failure stress (MPa) were computed by the system software (WaveMatrix 1.5, Instron, France).

### Statistical analysis

Data are shown as mean±SD with replicates being biological replicates. Statistical analysis was performed by applying analysis of variance and Kruskal-Wallis non-parametric test using Prism V.10 (GraphPad, USA). A p<0.05 was considered statistically significant.

## Results

### Distinct upregulation of IL-17A in human early-stage tendinopathy

**Figure 1:**
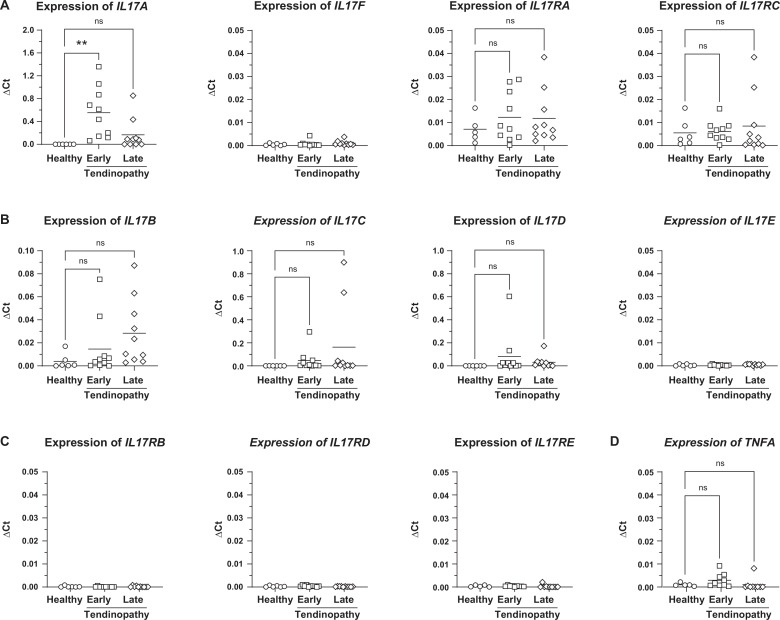
Expression of *IL17A* and its receptor heterodimer subunits *IL17RA* and *IL17RC* compared with other IL-17 family member ligands and receptors in tendon biopsies of human rotator cuff tendinopathy. (**A**) Expression of *IL17A, F* and *IL17RA, RC* (RT qPCR) (n=6–10 subjects). (**B**) Expression of *IL17B, C, D, E* (RT qPCR) (n=6–10 subjects). (**C**) Expression of *IL17RB, RD, RE* (RT qPCR) (n=6–10 subjects). (**D**) Expression of *TNFA* (RT qPCR) (n=6–10 subjects). The relative mRNA expression of the gene of interest was calculated by the ΔCT method relative to the GAPDH housekeeping gene. Ordinary ANOVA (Šídák’s multiple comparisons test) was applied for statistical analysis, ^ns^p>0.05, **p<0.01. Data are presented as individual values (symbol) and as mean (line). Data are presented as individual values (symbols) and mean (line). Healthy (subscapularis tendon, no rotator cuff tear, Bonar score 1); Early-stage tendinopathy (subscapularis tendon, rotator cuff tear of adjacent supraspinatus tendon, Bonar score 2–3); Late-stage tendinopathy (supraspinatus tendon with tear, Bonar score 4). ANOVA, analysis of variance; GAPDH, glyceraldehyde-3-phosphate dehydrogenase; IL17A to F, interleukin 17 A to F mRNA; IL17RA to RE, interleukin 17 receptor A to E mRNA; ns, not significant; TNFA, tumour necrosis factor alpha mRNA.

In the present study using RT qPCR, we demonstrate the mRNA expression of IL-17A (*IL17A*) in human early-stage tendinopathy (figure 1A), confirming our previous data.^10^ We found no evidence of *IL17F* expression (figure 1A). In addition, here, we demonstrate the differential expression of *IL17A* in contrast to the other IL-17 family members, *IL17B, IL17C, IL17D and IL17E* in early-stage tendinopathy compared with late-stage tendinopathy and healthy tendon (figure 1B). The IL-17A receptor heterodimer subunits *IL17RA* and *IL17RC* were constitutively expressed with medium abundance; however, *IL17RA* and *IL17RC* receptor expression was not linked to the upregulated expression of IL17A and was not significantly altered in tendinopathy (figure 1A, online supplemental figure 1). The receptor subunits *IL17RB*, *IL17RD* and *IL17RE* of the other IL-17 family members were not expressed (figure 1C). Moreover, TNF-α RNA (*TNFA*) was expressed at low abundance in early-stage tendinopathy; however, statistically significant differential expression was not apparent (figure 1D).

### IL-17A induces inflammatory gene signature in human tendon-derived cells

**Figure 2:**
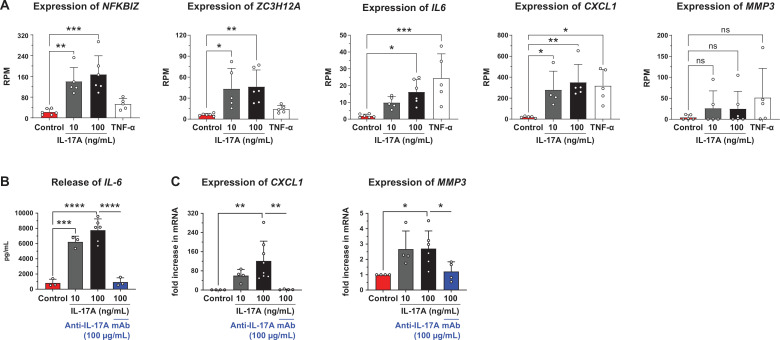
Expression of selected IL-17A pathway signature genes and inhibition thereof with the anti-IL-17A IgG_1_ mAb secukinumab in cells derived from healthy human hamstring and gracilis tendon. (**A**) Expression levels of *NFKBIZ, ZC3H12A, IL6, CXCL1* and *MMP3* measured by AmpliSeq RNA after stimulation with IL-17A and TNF-α (0.15 ng/mL) (n=4–6 subjects/1–3 hamstring and three gracilis tendons, in total three outlier data points (*CXCL1*) were identified and removed using ROUT with Q=1%). (**B**) Anti-IL-17A mAb secukinumab inhibition (100 µg/mL) of IL-17A stimulated release of IL-6 (ELISA) (n=3–6 subjects/hamstring tendons). (**C**) Anti-IL-17A mAb secukinumab inhibition (100 µg/mL) of IL-17A stimulated expression of *CXCL1* and *MMP3* (RT qPCR) (n=4–8 subjects/hamstring tendons, in total 2 outlier data points (*MMP3*) were identified and removed using ROUT with Q=1%). Ordinary ANOVA (Šídák’s multiple comparisons test) or Kruskal-Wallis test (Dunn’s multiple comparisons test) was applied for statistical analysis, *p<0.05, **p<0.01, ***p<0.001, ****p<0.0001. Data are presented as individual values (symbol) and as mean±SD (bar). ANOVA, analysis of variance; *CXCL1*, chemokine (C-X-C motif) ligand 1 mRNA; IL-17A, recombinant human interleukin 17A; mAb, monoclonal antibody; *NFKBIZ*, NF-kappa-B inhibitor zeta mRNA; ROUT, robust nonlinear regression and outlier removal; RPM, reads per million mapped reads; TNF-α, tumour necrosis factor alpha; *ZC3H12A*, zinc finger CCCH-type containing 12A mRNA.

According to the distinct upregulation of IL-17A in human early-stage tendinopathy, subsequent experiments focused on IL-17A and explored the regulation of the selected inflammatory IL-17A pathway signature genes, NFKBIZ (transcription factor NFKB inhibitor zeta), ZC3H12A (transcription factor Zinc Finger CCCH-Type Containing 12A), IL-6 (cytokine interleukin 6), CXCL1 (chemokine C-X-C motif ligand 1) and MMP3 (matrix metalloprotease 3) following IL-17A and TNF-α stimulation of healthy tendon-derived cells. IL-17A but not TNF-α upregulated the expression of the transcription factors *NFKBIZ* and *ZC3H12A* in cells derived from hamstring and gracilis tendons (figure 2A). Expression of the post-transcriptional effectors *IL6, CXCL1 and MMP3* was upregulated by both IL-17A and TNF-α. Of note, *MMP3* was abundantly expressed in cells derived from hamstring tendons but not in cells derived from gracilis tendons, resulting in high variability (figure 2A). The effect of IL-17A blockade was assessed on the post-transcriptional effectors because of their downstream integrated mediation of inflammation and degeneration in the pathogenesis of tendinopathy. The anti-IL-17A mAb secukinumab inhibited the IL-17A stimulated release of IL-6 (figure 2B) and expression of *CXCL1* and *MMP3* (figure 2C) in cells derived from hamstring tendons.

### IL-17A induces an inflammatory milieu and promotes tendon degeneration in rat tail tendon fascicles ex vivo

**Figure 3:**
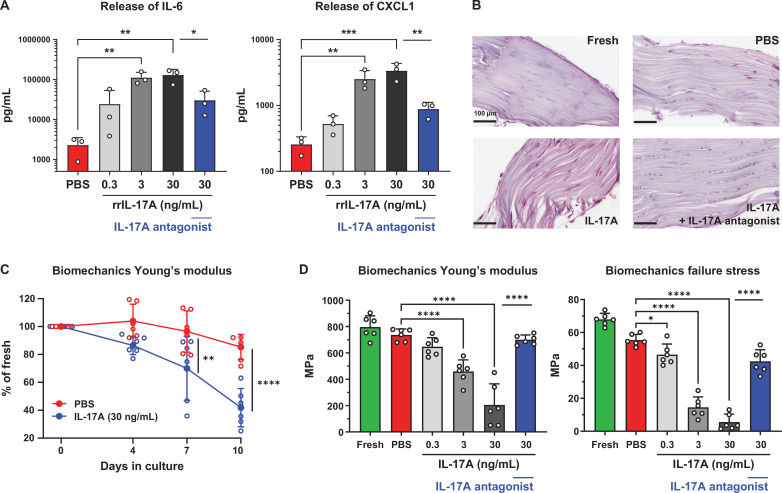
Reversal of IL-17A-mediated inflammatory mediator release, structural degeneration and functional impairment and reversal thereof with an IL-17A antagonist of isolated rat tail tendon fascicles ex vivo. (**A**) IL-17A antagonist inhibition (0.625 µg/mL) of IL-17A mediated protein release of CXCL1 and IL-6 in fascicle culture supernatant analysed by ELISA (n=3 rats). (**B**) Representative histological sections of tendon fascicles freshly harvested and at day 10 of culture with IL-17A (30 ng/mL) without and with IL-17A antagonist (1 µM) as well as with PBS (control). Paraffin-embedded tendon fascicles were cut along the longitudinal axis and 5 µm thick slices were obtained from the fascicle core and stained with Alcian blue and Nuclear Fast Red. (**C**) Time course of IL-17A-mediated degeneration measured as decline in fascicle mechanical stiffness (Young’s modulus) (n=6 rats). (**D**) IL-17A antagonist inhibition (0.625 µg/mL) of IL-17A mediated degeneration measured as decline in fascicle mechanical stiffness (Young’s modulus) and tensile strength (failure stress) of fascicles freshly harvested and at day 10 of culture with IL-17A and PBS (n=6 rats). Ordinary ANOVA (Šídák’s multiple comparisons test) was applied for statistical analysis, *p<0.05, **p<0.01, ***p<0.001, ****p<0.0001. Data are presented as individual values (symbol) and as mean±SD (bar). ANOVA, analysis of variance; CXCL1, chemokine (C-X-C motif) ligand 1; PBS, phosphate buffered saline; ROUT, robust non-linear regression and outlier removal.

Like in human tendon-derived cells, we explored the extent to which IL-17A can also induce an inflammatory milieu in the rat tail tendon fascicle assay. In tendon fascicles cultured without mechanical loading for 10 days, IL-17A significantly stimulated the release of CXCL1 and IL-6. Inhibition with the IL-17A antagonist significantly attenuated the release of these inflammatory effectors (figure 3A).

We then tested the effect of IL-17A on the structural and functional integrity of the tendon fascicles. In the absence of mechanical loading and in culture with IL-17A, the extracellular matrix of tendon fascicles appeared unravelled and disintegrated throughout their length and width. IL-17A augmented the tendon fascicle degeneration with a more profound phenotype of degenerative disorganisation of tendon extracellular matrix (figure 3B) and an augmented impairment of tendon fascicle mechanical function compared with control fascicles (figure 3C). The IL-17A antagonist prevented the IL-17A-induced augmentation of degenerative disorganisation of tendon extracellular matrix and attenuated the impairment of tendon fascicle mechanical stiffness (Young’s modulus) and tensile strength (failure stress) (figure 3D).

### IL-17A blockade attenuates inflammation and restores impaired tendon function in rat rotator cuff tendinopathy in vivo

We finally explored the immunomodulatory and functional relevance of IL-17A blockade in vivo in a rat model of rotator cuff tendinopathy.

**Figure 4:**
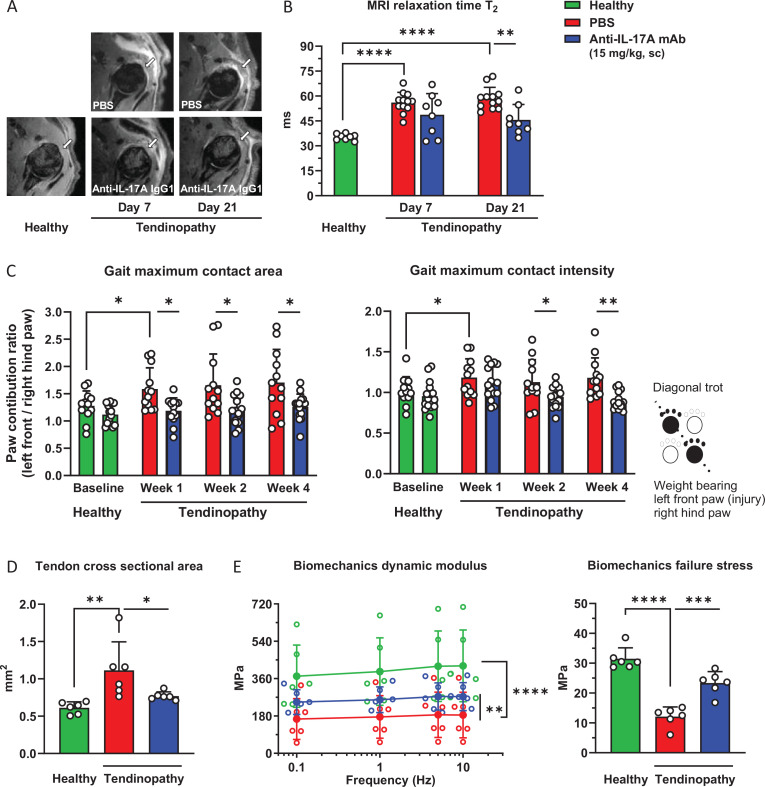
Effects of IL-17A blockade with the anti-IL17A IgG1 mAb on inflammation, gait imbalance and tendon mechanical function in a rat disease model of rotator cuff tendinopathy in vivo. (**A**) Representative MRI of healthy and injured rotator cuff at day 7 and day 21 postinjury and weekly subcutaneous dosing of anti-IL17A mAb and PBS (control). Arrows indicate the anatomical location of the supraspinatus tendon. (**B**) Anti-IL-17A mAb reversal of rotator cuff inflammation quantified by MRI relaxation time T_2_ (n=6–12 rats). (**C**) Anti-IL-17A mAb reversal of shoulder dysfunction assessed by gait analysis of paw maximum contact area and maximum paw contact intensity (n=12–16 rats, in total four outlier data points were identified and removed using ROUT with Q=1%). (**D**) Anti-IL-17A mAb attenuation of supraspinatus tendon swelling assessed as tendon cross-sectional area (n=6 rats). (**E**) Anti-IL-17A mAb attenuation of supraspinatus tendon impaired mechanical stiffness (dynamic modulus) and tensile strength (failure stress) (n=6 rats). Ordinary and two-way ANOVA (Šídák’s, Tukey’s and Dunnett’s multiple comparisons test) was applied for statistical analysis, *p<0.05, **p<0.01, ***p<0.001, ****p<0.0001. Data are presented as individual values (symbol) and as mean±SD (bar). ANOVA, analysis of variance; IL-17A, interleukin 17A; T_2_, relaxation time; mAb, monoclonal antibody; PBS, phosphate buffered saline; sc, subcutaneous.

Tendon inflammation was quantitatively assessed by increased T_2_-weighted signal in MRI scans. The anti-inflammatory effect of IL-17A blockade on acute and subacute tendon inflammation was longitudinally measured up to 3 weeks after supraspinatus tendon injury (figure 4A). Sustained injury-induced tendon inflammation was demonstrated by the significantly increased MRI T_2_ signal for up to 3 weeks after injury (figure 4B). Treatment with the anti-IL-17A mAb had no significant anti-inflammatory effect on the early acute phase inflammation. However, the anti-IL-17A mAb resolved inflammation and reversed the increased MRI T_2_ signal back to baseline values during the sustained sub-acute inflammatory phase (figure 4B). In line with these results, geometric size assessment of terminally excised supraspinatus tendons 4 weeks postinjury revealed reversal of tendon swelling after anti-IL-17A mAb treatment as indicated by normalisation of tendon cross-sectional area (figure 4D).

Gait analysis was performed longitudinally as an integrated assessment of shoulder function to investigate the potential therapeutic benefit of IL-17A blockade up to 4 weeks after supraspinatus tendon injury. Supraspinatus tendon injury induced a sustained shoulder function deficit indicated by gait imbalance in the rat weight-bearing diagonal trot and significantly increased left front—right hind paw ratios of maximum contact area and contact intensity up to 4 weeks after injury. The anti-IL-17A mAb reversed the impaired shoulder function over time, with consistent significant improvement versus vehicle starting 2 weeks postinjury (figure 4C).

Mechanical testing of terminally excised supraspinatus tendons was applied to assess the direct effect of IL-17A blockade on tendon function 4 weeks postinjury. The anti-IL-17A mAb significantly improved the impaired tendon mechanical function, demonstrated by increased supraspinatus tendon stiffness (dynamic modulus) and tensile strength (failure stress) (figure 4E).

## Discussion

It has previously been demonstrated that in tendon biopsies of human tendinopathy, IL-17A mRNA (*IL17A*) and IL-17A protein are upregulated.^10 18^ Here, we describe that only *IL-17A,* and no other IL-17 family member is differentially expressed in rotator cuff tendon biopsies of human early-stage tendinopathy. In contrast, in human late-stage tendinopathy, we have not observed differential expression of *IL17A* and the other IL-17 family members. These findings correspond with global gene expression and single-cell transcriptomics in tendon biopsies obtained from patients with end-stage chronic tendinopathy, suggesting that inflammatory immune effectors are differentially regulated in different stages of tendinopathy.^7 19^ We also found that IL-17F, which can form an active IL-17A/IL-17F heterodimer, is not expressed in rotator cuff tendon biopsies, in line with recent findings showing that healthy and diseased human tendon-derived fibroblasts are responsive to IL-17A but not to IL-17F to induce intracellular signalling and mRNA expression of inflammatory immune effectors.^18^ We demonstrate that only the receptor subunits *IL17RA and IL17RC* through which IL-17A and IL-17F signals are expressed in rotator cuff tendon biopsies. However, *IL17RA and IL17RC* receptor expression is not linked to *IL17A* expression and is not regulated in tendinopathy. Together these findings suggest that upregulation of the receptor ligand IL-17A is the key driver to boost signal induction in tendinopathy and that IL-17A is the main IL-17 cytokine family member contributing to the pathogenesis of human early-stage tendinopathy.

Future work may nevertheless investigate other IL-17 family members, for example, IL-17B in late-stage tendinopathy. To appropriately address the immensely important question of ‘personalised’ molecular pathology and prediction of therapy outcome, we propose well-powered single-cell resolution spatial transcriptomic studies encompassing all stages of tendinopathy.

In subsequent experiments in healthy human tendon-derived cells, we, therefore, focused on IL-17A and the upregulation of the selected IL-17A pathway signature genes *NFKBIZ, ZC3H12A, IL6, CXCL1* and *MMP3*. This gene selection was made based on evidence generated in many inflammatory conditions and in various cell types including human tendon-derived fibroblasts demonstrating IL-17A to signal through the NF-κB complex and to selectively induce the transcription factors NFKBIZ and ZC3H12A and the downstream inflammatory immune effectors IL-6, CXCL1 and MMP3.^20^,^23^ Here, we show in healthy human tendon-derived cells that the expression of *NFKBIZ* and *ZC3H12A* was upregulated by IL-17A but not by TNF-α. In contrast, *IL6*, *CXCL1* and *MMP3* were upregulated by both IL-17A and TNF-α. The IL-17A-induced expression of downstream inflammatory effectors IL-6, *CXCL1* and *MMP3* was completely inhibited by secukinumab, suggesting that IL-17A alone and in concert with TNF-α may promote inflammation in tendinopathy.

In the present work, we induced IL-17A pathway activation in cells derived from healthy tendons as a mechanistic experimental model to mimic the suggested IL-17A-mediated pathogenesis of tendinopathy. Future work is warranted to confirm IL-17A pathway activation in cells derived from diseased tendinopathic tendons. For future work, it is also proposed to use cells derived from healthy tendons of the same anatomical origin, ideally matching the anatomical site of the diseased tendon. Although tendon progenitor cells and tenocytes are considered to represent 90%–95% of the midsubstance cell population of healthy tendons,^24^ the local environment of tendons, such as blood supply and mechanical load, varies between different anatomical sites and may influence tendon cellular composition and tenocyte cellular function. In fact, recent human tendon single-cell transcriptomics identified distinct tenocyte subpopulations.^7^

Tendinopathy and enthesitis have been proposed to share pathogenetic commonalities.^11^,^13^ In spondyloarthritis and psoriatic arthritis, enthesitis is a characteristic clinical manifestation, and anti-IL-17A and anti-TNF-α therapies have shown therapeutic efficacy indicating an important role of IL-17A and TNF-α in tendo-enthesial pathologies.^15 25 26^ In tendinopathy, today only one anti-IL-17A clinical Ph2 trial was performed to assess efficacy, safety and tolerability of secukinumab in a broad patient population encompassing patients with early-stage and late-stage rotator cuff tendinopathy (NCT03344640). This study—while it failed its primary endpoint in the broad study population—revealed significant and clinically relevant improvement of tendinopathic symptoms in patient-reported outcome measures (PROs) by anti-IL-17A treatment versus placebo in the patient population with early-stage moderate-to-severe rotator cuff tendinopathy.^16^

By using the ex vivo rat tendon fascicle model and the in vivo rat rotator cuff tendinopathy model, we aimed to further elucidate the biological mechanisms of IL-17A blockade underlying the reported therapeutic effects in human early-stage tendinopathy. Of note, in the current work, validation of the rat models regarding expression of IL-17 family members and their receptor subunits was not performed. For future work, it is proposed to conduct a dedicated transcriptomic study on *IL-17* ligand and receptor expression in the rat model of rotator cuff tendinopathy and to investigate in the model the effect of IL-17A blockade on differential gene expression, ideally using single-cell resolution spatial transcriptomics.

These functional rat ex vivo and in vivo tendinopathy models have been selected because of their representation of clinical, histological, cellular and molecular features of human tendinopathy, such as tendon swelling, impaired mechanical function, loss of extracellular matrix integrity, immune cell infiltration and upregulated expression of immune effectors such as alarmins, cytokines, chemokines and matrix metalloproteinases.^427^,^29^ Indeed, in the isolated rat tendon fascicle assay, we demonstrate that IL-17A stimulated the release of the selected post-transcriptional inflammatory effectors IL-6 and CXCL1 and induced phenotypes of tendon degeneration and tendon impaired mechanical function, all of which were significantly attenuated by IL-17A blockade. The observed phenotype of tendon degeneration is proposed to be linked with IL-17A-induced downstream upregulation of matrix metalloproteinase expression, which may disrupt extracellular matrix homeostasis due to augmented matrix protein degradation.^18 30^ Our mechanistic data generated in tendon fascicles suggest that blockade of IL-17A signal induction controls the downstream inflammatory IL-17A signalling cascade and may prevent progression of tendon degeneration in tendinopathy.

Immune modulatory effects of IL-17A blockade became also apparent in our in vivo rat disease model of rotator cuff tendinopathy. We observed that the anti-IL-17A mAb accelerated the resolution of active inflammation in response to supraspinatus tendon injury. The concomitantly observed improvement in tendon and shoulder function indicates that the biological mechanism of action of IL-17A blockade has the potential to interfere with the pathobiology of tendinopathy. Importantly, in combination, these findings suggest that IL-17A blockade does not offset or dysregulate the acute phase inflammatory response, which is key for physiological tissue healing and regeneration.^31^ Moreover, these findings also suggest that in tendinopathy, IL-17A blockade does not interfere with alarmin signalling. Noteworthy, alarmins, the key mediators of acute phase inflammation, have been reported to be upregulated in human tendinopathy^32^,^36^ and in rodent models of tendinopathy on tendon injury.^37 38^

In our in vivo rat disease model of rotator cuff tendinopathy, we were attentive but not successful in exploring potential antinociceptive effects of IL-17A blockade on tendinopathic pain, another hallmark of tendinopathy. The applied CatWalk gait assessment has previously been reported to be suitable for the investigation of chronic pain-related behaviour in rat models of severe trauma such as unstabilised rotator cuff rupture and progressing arthritis by demonstrating a pain-related imbalance in weight-bearing of the diseased ipsilateral and the healthy contralateral paw.^39 40^ However, data generated in less severe rat models of rotator cuff tendon tear and repair^39 41^ informed about the potential limitations of gait analysis for studying tendinopathic pain. Correspondingly, in our low severity rat model of rotator cuff tendinopathy, we have not observed pain-related gait imbalances comparing the injured ipsilateral and the healthy contralateral paw (data not shown). The reasoning that these inherent experimental limitations may have masked the preclinical effect of IL-17A blockade on tendinopathic pain is also supported by the preliminary explorative clinical data on secukinumab, providing significant and fast-onset relief of tendinopathic pain in patients with early-stage rotator cuff tendinopathy.^16^

Another inherent experimental limitation of our studies concerns the IL17-A ligand neutralisation in the in vitro and ex vivo pharmacology experiments. We demonstrated that neutralising the IL-17A ligand prevents pathway signalling and related downstream biological effects; however, one should note that binding affinity and binding kinetics of the inhibitors to the IL-17A ligand determined the overall pharmacological response in these experiments.

Taken together, we provide evidence supporting the hypothesis that IL-17A is a key contributor to the pathogenesis of human rotator cuff tendinopathy. We show that IL-17A stimulates signalling cascades that promote tendon inflammation and tendon matrix degeneration, proposed to be causally linked with the clinical manifestation of pain and impaired tendon function in tendinopathy. In addition, in a rat model of rotator cuff tendinopathy, we provide evidence that IL-17A blockade with an anti-IL-17A mAb leads to a reversal of tendon tissue inflammatory and degenerative changes and to the restoration of impaired tendon function, proposed to be the underlying biological mechanisms of the clinical symptom relief by secukinumab in human early-stage tendinopathy. IL-17A blockade may, therefore, be a potential therapy in early-stage tendinopathy.

## supplementary material

10.1136/rmdopen-2024-004729 online supplemental figure 1

## Data Availability

Data are available on reasonable request.
