# Supplementary material for: Targeting the IL-17A pathway for therapy in early-stage tendinopathy
Source: RMD Open. 2025 Feb 23;11(1):e004729. doi: 10.1136/rmdopen-2024-004729 (PMC11881027; doi:10.1136/rmdopen-2024-004729)
Supplement: online supplemental figure 1 [file rmdopen-11-1-s001.docx]

**Supplementary Figure 1**

**Expression and correlation of *IL17A* with its receptor heterodimer subunits *IL17RA* and *IL17RC* and with the IL-17 family member *IL17B* in *IL17A* high and low expressing subscapularis tendon biopsies (early-stage tendinopathy)**

**
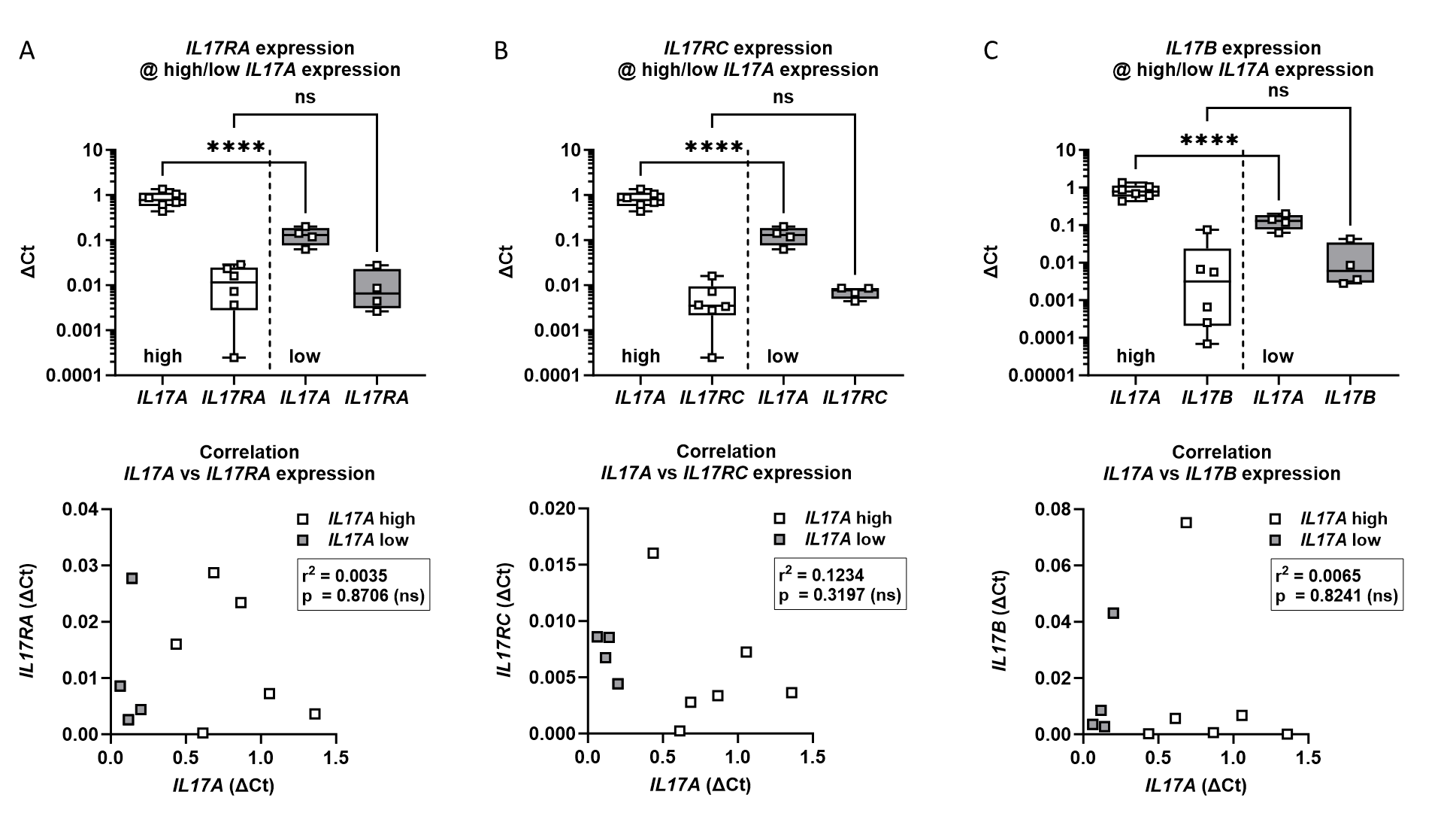
**

**(A) Expression and correlation of *IL17A* and *IL17RA* (RT qPCR). (B) Expression and correlation of *IL17A* and *IL17RC* (RT qPCR). (C) Expression and correlation of *IL17A* and *IL17B* (RT qPCR).**

**The relative mRNA expression of the gene of interest was calculated by the ΔCT method relative to the GAPDH housekeeping gene.**

**Ordinary ANOVA (Šídák's multiple comparisons test) and Pearson correlation calculation was applied for statistical analysis, ^ns^ p > 0.05, ****p<0.0001.**

**Data are presented as individual values (symbol) and as median with min to max range and 10 to 90 percentiles (box) derived from n = 6 subjects with *IL17A* high expression and n = 4 subjects with *IL17A* low expression.**

**GAPDH, Glyceraldehyde-3-phosphate dehydrogenase; *IL17A,* interleukin 17 A mRNA; *IL17B,* interleukin 17 B mRNA; *IL17RA,* interleukin 17 receptor A mRNA;** ***IL17RC,* interleukin 17 receptor C mRNA**

**Early-stage tendinopathy (subscapularis tendon, rotator cuff tear of adjacent supraspinatus tendon, Bonar score 2-3).**
